# Can Ceylon Leadwort (*Plumbago zeylanica* L.) Acclimate to Lead Toxicity?—Studies of Photosynthetic Apparatus Efficiency

**DOI:** 10.3390/ijms21051866

**Published:** 2020-03-09

**Authors:** Krzysztof M. Tokarz, Wojciech Makowski, Barbara Tokarz, Monika Hanula, Ewa Sitek, Ewa Muszyńska, Roman Jędrzejczyk, Rafał Banasiuk, Łukasz Chajec, Stanisław Mazur

**Affiliations:** 1 Department of Botany, Physiology and Plant Protection, Faculty of Biotechnology and Horticulture, University of Agriculture in Krakow, Al. 29 Listopada 54, 31-425 Kraków, Poland; wojtek.makowski.1305@gmail.com (W.M.); barbara.tokarz@urk.edu.pl (B.T.); monika.hanula@sggw.pl (M.H.); ewa.sitek@urk.edu.pl (E.S.); stanislaw.mazur@urk.edu.pl (S.M.); 2 Department of Technique and Food Development, Institute of Human Nutrition Sciences, Warsaw University of Life Sciences, Nowoursynowska 159 C, 02-776 Warsaw, Poland; 3 Department of Botany, Institute of Biology, Warsaw University of Life Sciences, Nowoursynowska 159/37, 02-776 Warsaw, Poland; ewa_muszynska@sggw.pl; 4 Plant-Microorganism Interactions Group, Malopolska Centre of Biotechnology, Jagiellonian University, Gronostajowa 7A, 30-387 Kraków, Poland; roman.jedrzejczyk@uj.edu.pl; 5 Institute of Biotechnology and Molecular Medicine, Trzy Lipy 3, 80-172 Gdansk, Poland; banasiuk@gmail.com; 6 Institute of Biology, Biotechnology and Environmental Protection, Faculty of Natural Sciences, University of Silesia, Bankowa 9, 40-007 Katowice; lukasz.chajec@us.edu.pl

**Keywords:** alternative electron transport, antioxidant enzymes, Chl fluorescence, heavy metals, photosynthesis, secondary metabolites

## Abstract

Ceylon leadwort (*Plumbago zeylanica*) is ornamental plant known for its pharmacological properties arising from the abundant production of various secondary metabolites. It often grows in lead polluted areas. The aim of presented study was to evaluate the survival strategy of *P. zeylanica* to lead toxicity via photosynthetic apparatus acclimatization. Shoots of *P. zeylanica* were cultivated on media with different Pb concentrations (0.0, 0.05, and 0.1 g Pb∙l^−1^). After a four-week culture, the efficiency of the photosynthetic apparatus of plants was evaluated by Chl *a* fluorescence measurement, photosynthetic pigment, and Lhcb1, PsbA, PsbO, and RuBisCo protein accumulation, antioxidant enzymes activity, and chloroplast ultrastructure observation. Plants from lower Pb concentration revealed no changes in photosynthetic pigments content and light-harvesting complex (LHCII) size, as well as no limitation on the donor side of Photosystem II Reaction Centre (PSII RC). However, the activity and content of antioxidant enzymes indicated a high risk of limitation on the acceptor side of Photosystem I. In turn, plants from 0.1 g Pb∙l^−1^ showed a significant decrease in pigments content, LHCII size, the amount of active PSII RC, oxygen-evolving complex activity, and significant remodeling of chloroplast ultrastructure indicated limitation of PSII RC donor side. Obtained results indicate that *P. zeylanica* plants acclimate to lead toxicity by Pb accumulation in roots and, depending on Pb concentration, by adjusting their photosynthetic apparatus via the activation of alternative (cyclic and pseudocyclic) electron transport pathways.

## 1. Introduction

The intensive development of metallurgy, ore mining, chemical and fertilizer industry, municipal services, and other industries involves the production of various waste types that increase environmental pollution. Among most hazardous wastes are heavy metals [1,2]. Despite many attempts to withdraw heavy metals from use, complete elimination of the problem associated with the emission of these elements into the environment is still not possible [1]. The most toxic heavy metals that threaten human health are cadmium, lead, arsenic, and mercury [3]. People are exposed to them through different pathways like air, household dust, street dirt, soil, water and food, including the consumption of plants growing in polluted areas [4].

Lead is a threat not only to people but also exerts a negative effect on plants [5]. Visible symptoms of lead toxicity to plants are manifested by retardation of the growth and development of plant organs and the reduction of plant yield [5,6,7,8]. These reductions result mainly from oxidative stress, photosynthesis inhibition, and damage to DNA and its consequences caused by Pb ions [9]. Although lead is classified as a metal with low redox activity, i.e., it cannot generate ROS in a direct oxidoreductive reaction such as the Haber–Weiss/Fenton reaction, it still increases ROS in the cells. Lead causes emergence of ROS by stimulation of NADPH oxidase, substitution of divalent cations at enzyme cofactors, limitation of enzyme activity by binding to -SH or -COOH groups, or peroxidation of lipids [10,11]. Moreover, lead decreases activity of antioxidant enzymes by interference with the effective accumulation of K, P, Fe, Zn, Cu, Mg and Ca elements [12]. Pb may restrain photosynthesis by reducing chlorophyll synthesis (associated with ALAD activity), interfering with electron transport within Photosystem II (PSII) and Photosystem I (PSI), as well as inhibiting plastoquinone synthesis, damaging oxygen-evolving complex (OEC), or lowering activity of Calvin-Benson cycle enzymes [13,14]. The light-independent reactions of photosynthesis (“dark reactions”) seems to be very sensitive to Pb which significantly decreases CO_2_ assimilation rate and activity of RuBisCo [15]. Lead genotoxicity results from its ability to binding directly or indirectly (by proteins) to DNA and disrupting DNA replication or repair [16]. Moreover, Pb disturbs mitotic activity of cells, very often leading to c-mitosis [17]. Disruptions of mitosis may also result from Pb interference with the cytoskeleton, which leads to chromosome aberrations [18].

First reaction of plants to the lead presence in soil is metal retention on the root surface and/or selective metal collection [13]. After entering into cells, lead is neutralized by accumulation in inter-cell spaces, cell walls (binding to pectin carboxyl groups), vacuoles, and in small amounts in dictyosomes and the endoplasmatic reticulum [10,14,19,20]. Thanks to these mechanisms lead is mainly accumulated in the roots and only a small amount of lead is transported to plant shoots, most likely because of the physical barrier of Casparian strips in endoderm cells [13]. Nevertheless, some amounts of Pb ions entering the cells trigger the plant’s defense mechanisms based mainly on antioxidant system (enzymes and small-molecule antioxidant) [5,8]. Moreover, in the case of Pb penetration to the aboveground plant cells, plant survival will be determined by the ability to acclimatize the photosynthetic apparatus to the existing conditions [20].

Ceylon leadwort (*Plumbago zeylanica* L.) is a decorative plant known for its pharmacological properties resulting from the abundant production of various secondary metabolites, among which the most-known is plumbagin (a derivative of 1,4-naphthoquinone) [21]. Ceylon leadwort belongs to Plumbaginaceae family known from species, like sea thrift (*Armeria maritime*) and cape leadwort (*Plumbago auriculata* Lam) often found in lead polluted regions [22]. The *Plumbago* genus name that originated from Latin words *plumbum* (“lead”) is believed to refer to lead-blue flower colour, the ability of the sap to create lead-colored stains on skin, or the belief that the plant was a cure for lead poisoning [23]. The natural place of *Plumbago* occurrence is South Asia, especially India. Moreover, it can be found in subtropical and tropical regions above 2000 m above sea level, where soils are rich in trace elements [24]. All this suggests that *Plumbago* plants are associated with lead and may be tolerant to this harmful element. However, there are no papers on the effect of lead ions on *P. zeylanica*, an otherwise very important pharmacological species.

Therefore, presented research was focused on the response of *P. zeylanica* to lead toxicity. Our aim was to evaluate the plants survival strategy mainly in the context of photosynthetic apparatus acclimatization. Experiments were conducted under controlled in vitro conditions to exclude malfunctions of plant metabolism caused by other adverse factors, such as drought, high light intensity, pathogens, etc.

Presented research indicates that *P. zeylanica* plants acclimate to lead toxicity by adjusting their photosynthetic apparatus via different mechanisms (alternative electron transport pathways) depending on Pb ion concentration.

## 2. Results

### 2.1. Growth and Development of P. zeylanica

**Figure 1:**
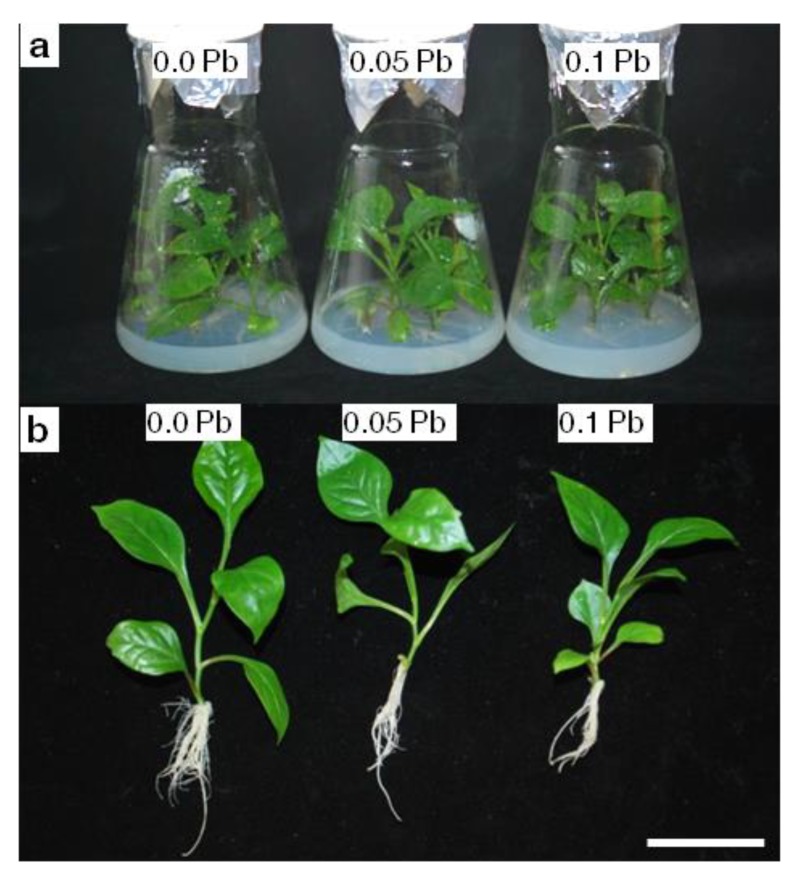
*In vitro* culture (**a**) and plants (**b**) of Ceylon leadwort (*P. zeylanica*) after 4 weeks of culture on the media with different Pb concentration. Scale bar, 5 cm.

First symptom of lead toxicity is reduction of plant size and biomass [8,13]. To evaluate effect of lead on *P. zeylanica* growth and development, estimation of the fresh shoot mass increase in relation to the final fresh shoot mass (shoot growth index), rooting efficiency, and dry weight of organs were measured. After 4 weeks of *P. zeylanica* culture no statistically significant differences were noted in shoot growth index on media containing different concentrations of Pb (Figure 1a,b, Table 1). While, in presented study it was observed that roots are more vulnerable to lead toxicity. Pb affected rooting efficiency, both rooting percentage and root length (Table 1). Nonetheless, rooting percentage was reduced only on the medium with 0.1 g Pb∙l^−1^ but average root length decreased in presence of both Pb concentrations applied. However, media supplemented in Pb did not influence root dry weight content, but shoot dry weight content increased in the medium containing the highest Pb concentration (Table 1). It seems that analyzed plants in terms of growth parameters are not very susceptible to Pb ions.

### 2.2. Lead Concentration in Shoots and Roots

**Figure 2:**
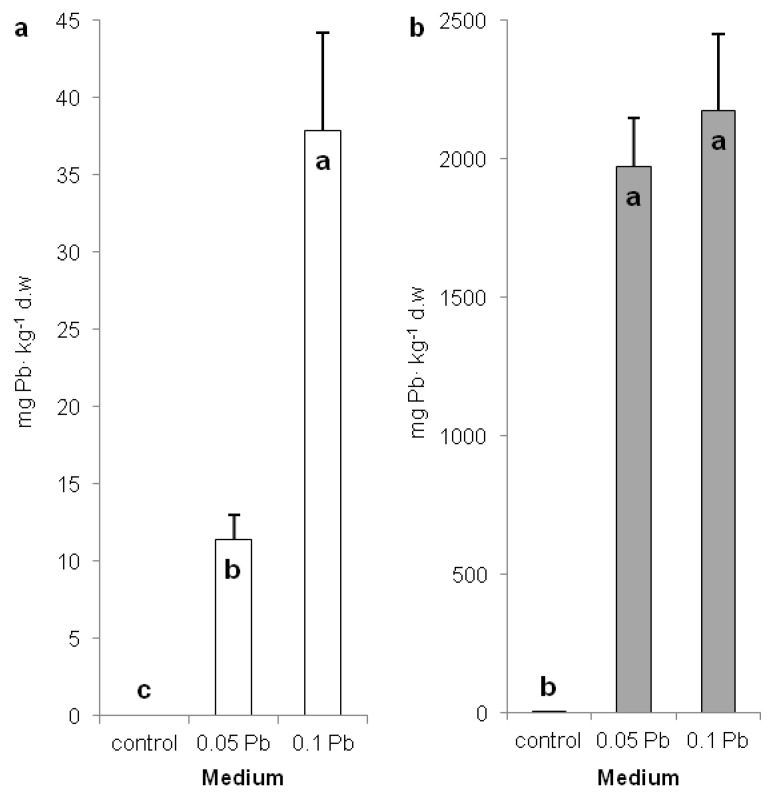
Lead content in shoots (**a**) and roots (**b**) of Ceylon leadwort (*Plumbago P. zeylanica*) depending on Pb concentration in the medium after 4 weeks of culture (different letters—statistically significant difference within each organ at *p* ≤ 0.05).

Assessment of the content of lead in different plant organs indicate the lead accumulation mode. *P. zeylanica* accumulated significantly more lead in shoots of plants cultivated on medium with 0.1 g Pb∙l^−1^ (38 mg∙kg^−1^ DW) than on medium with 0.05 g Pb∙l^−1^ (11 mg∙kg^−1^ DW) (Figure 2a). Meanwhile, lead accumulation was hundreds of times higher (1970 and 2174 mg∙kg^−1^ DW) in the roots but no statistical differences were observed between applied Pb doses (Figure 2b). The obtained results suggest that *P. zeylanica* is not a hyperaccumulator plant [25].

### 2.3. The Level of Cell Membranes Damage

Malondialdehyde (MDA) is a biomarker for the estimation of lipid-membranes peroxidation in oxidative stress [26]. In *P. zeylanica* plants MDA accumulation significantly increased in shoots after 4 weeks of culture on medium supplemented with 0.1 g∙l^−1^ Pb (Table 2). In turn, MDA content did not change in roots regardless of the Pb concentration used (Table 2). This suggest that Pb ions accumulated in shoot cells, evoked significant oxidative stress, which, contrary to root cell response, were not excluded by shoot cell defense mechanisms.

### 2.4. Accumulation of Protecting Secondary Metabolites

Phenolic compounds content in plants changed under Pb stress (Table 2). In shoots of *P. zeylanica* content of phenolic compound significantly decreased with increased concentration of Pb in the medium. In turn, roots of examined plants accumulated significantly more phenolic compounds on media with lead. After 4 weeks of culture on media containing Pb, cinnamic acid derivatives accumulation did not change in leadwort shoots (Table 2). In contrast, cinnamic acid derivatives accumulation in roots decreased with an increased dose of Pb in the medium. Flavonols content decreased in shoots of *P. zeylanica* on medium with 0.1 g Pb∙l^−1^, while in roots on media with both applied Pb doses (Table 2). Anthocyanin accumulation generally decreased in plants (shoots and roots) growing on media containing Pb. The exception was shoots from medium with 0.05 g Pb∙l^−1^, in which anthocyanin accumulation increased (Table 2). Plumbagin accumulation decreased in roots of plants cultured on media with Pb, but did not change in shoots of these plants (Table 2).

### 2.5. Effect of Lead on Photosynthetic Apparatus Efficiency

#### 2.5.1. Photosynthetic Pigments’ Content

**Figure 3:**
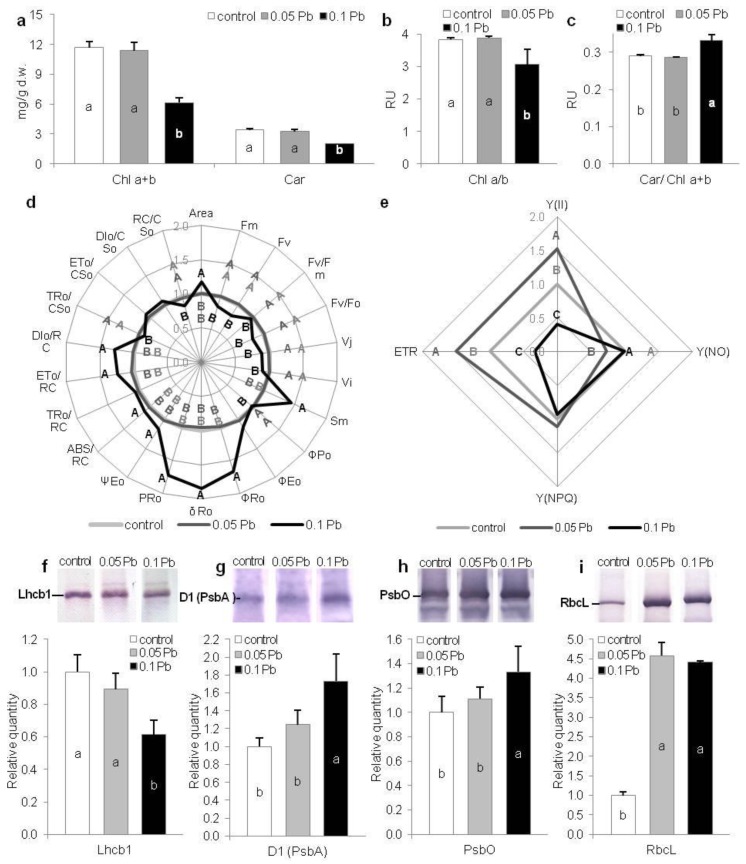
Photosynthetic apparatus efficiency of Ceylon leadwort (*P. zeylanica*) plants depending on Pb concentration in the medium after 4 weeks of culture; (**a**) chlorophylls and carotenoids content; (**b**) Chl *a*/*b* ratio; **c**) Car/Chl *a*+*b* ratio; (**d**) extracted and calculated fast kinetics Chl *a* fluorescent parameters; e) slow kinetics Chl *a* fluorescence parameters; (**f**–**h**) photosynthetic proteins’ content (**e**) Lhcb1; (**f**) D1(PsbA); (**g**) PsbO; (**h**) RcbL (different letters—statistically significant difference within each parameter at *p* ≤ 0.05; (**d**,**e**) values are given in relation to control (set as 1); abbreviations—see Table 3; RU—relative units).

To estimate condition of photosynthetic apparatus in *P. zeylanica* plants growing under Pb stress, content of photosynthetic pigments have been assayed. After 4 weeks of plants cultivation, a statistically significant decrease of total chlorophyll and carotenoids content together with simultaneous decrease of Chl *a*/*b* ratio was recorded in plants cultured on media with higher Pb concentration (0.1 g·l^−1^) (Figure 3a,b). Contrary, in the same conditions, Car/Chl *a*+*b* ratio significantly increased comparing to control conditions (Figure 3c). Chlorophyll and carotenoids contents as well as pigments ratios of plants from 0.05 g Pb·l^−1^ was on the same level as in control plants (Figure 3a–c).

#### 2.5.2. Chlorophyll *a* Fluorescence

Chl *a* fluorescence measurement enable assessment of PSII photochemistry efficiency and is widely used as an indicator of plant conditions under different abiotic stresses [27,28,29,30]. Generally, there were no differences between fast kinetics Chl *a* fluorescence parameters of plants cultivated on the media with 0.05 g Pb·l^−1^ and control plants (Figure 3d). In contrast, plants exposed to higher Pb concentration (0.1 g Pb·l^−1^) exhibited changed Chl *a* fluorescence parameters. Fluorescence parameters (maximum and variable fluorescence) declined in these plants (Figure 3d). Maximum quantum yield of PSII (F_V_/F_M_) and activity of water-splitting complex (Fv/F_0_) were also reduced in comparison to control plants. While reduced plastoquinone pool (Area) and total electron carriers per reaction center (RC) (Sm) increased. Also, parameters related to the electron flows (ψ_Eo,_ ρ_Ro,_ δ_Ro_) and quantum yield for the reduction of end acceptors of PSI per photon absorbed (φ_Ro_) increased in plants exposed to higher concentration of lead ions. Moreover, specific fluxes per RC (ABS/RC, ET_0_/RC, DI_0_/RC) increased but trapped energy flux per cross section (CS) (TR_0_/CS_0_) and amount of active PSII RCs per CS (RC/CS_0_) decreased (Figure 3d). Contrary, slow kinetics fluorescence parameters showed significant changes between control plants and plants from lower Pb concentration in the medium (0.05 g·l^−1^) (Figure 3e). Quantum photosynthetic yield of PSII—Y(II) increased in 0.05 g Pb·l^−1^ plants but decreased in 0.1 g Pb∙l^−1^ plants. While, all other non-photo-protective non-photochemical quenching—Y(NO) decreased in 0.05 g Pb·l^−1^ plants and did not change in 0.1 g Pb·l^−1^ plants (Figure 3e). In turn, photoprotective non-photochemical quenching—Y(NPQ) did not vary between examined plants from different conditions (Figure 3e). Moreover, electron transport rate (ETR) grew in 0.05 g Pb·l^−1^ plants and decreased in 0.1 g Pb∙l^−1^ plants (Figure 3e).

#### 2.5.3. Key Photosynthetic Proteins

Quantitative participation of photosystem proteins (Lhcb1, D1 (PsbA), PsbO) and RuBiSco was estimated by SDS-PAGE and immunobloting in shoots of *P. zeylanica* plants. The level of Lhcb1 decreased in plants treated with 0.1 g Pb·l^−1^ (Figure 3f), while in the same conditions, the quantity of D1 and PsbO proteins significantly increased compared to control plants (Figure 3g–i). Higher accumulation of RbcL was also noted in *P. zeylanica* shoots from media containing Pb (both concentrations) in comparison to control (Figure 3i).

#### 2.5.4. Leaves’ Anatomy and Chloroplasts’ Ultrastructure

**Figure 4:**
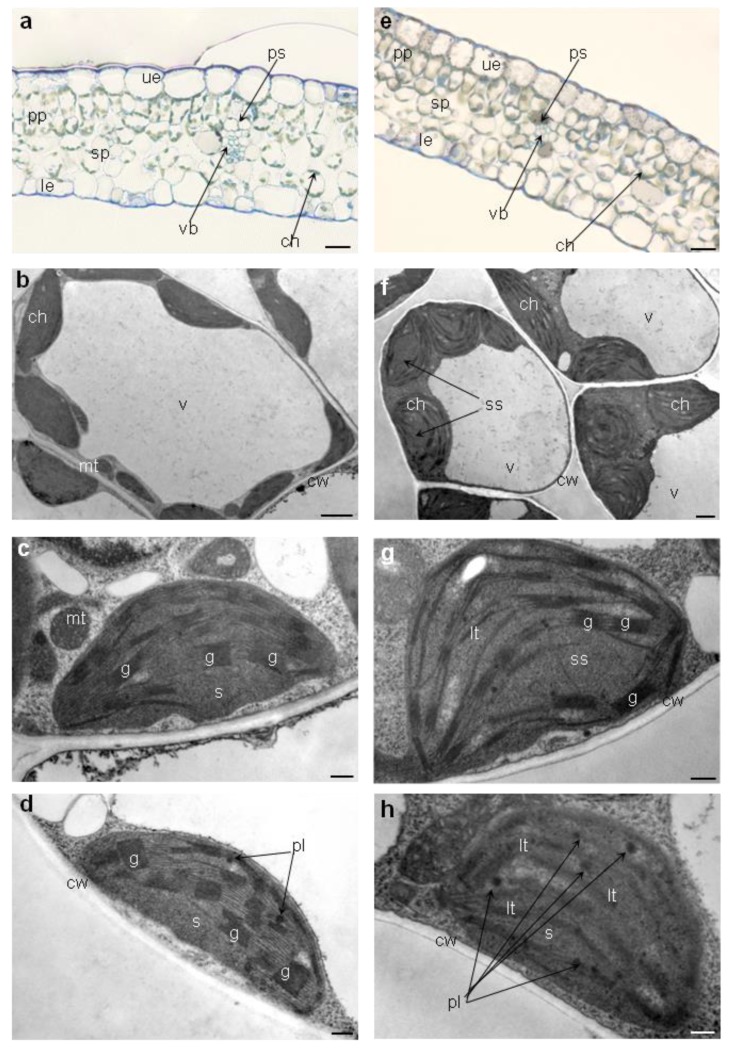
Leaf anatomy and chloroplasts ultrastructure of Ceylon leadwort (*P. zeylanica*) after 4 weeks of culture on the media with different Pb concentration; (**a**) anatomy of leaf from control medium; (**b**) mesophyll cell of leaf from control medium; c) ultrastructure of chloroplasts from control medium d) ultrastructure of chloroplasts from medium with 0.05 g Pb∙l^−1^; (**e**) anatomy of leaf from medium with 0.1 g Pb∙l^−1^; (**f**) mesophyll cell of leaf from medium with 0.1 g Pb∙l^−1^; (**g**) and h) ultrastructure of chloroplasts from medium with 0.1 g Pb∙l^−1^. Abbreviations: ue—upper epidermis, le—lower epidermis, pp—palisade parenchyma, sp—spongy parenchyma, ps—parenchyma sheath, vb—vascular bundle, ch—chloroplast, mt—mitochondrion, v—vacuole, cw—cell wall, s—stroma, ss—swollen stroma, g—grana, lt—loose thylakoids, pl—plastoglobule. Scale bars, 50 µm (**a**,**e**), 5 µm (**b**,**f**), 1 µm (**c**,**d**,**g**,**h**).

Generally, leaf anatomy of plants exposed to Pb toxicity did not vary from leaves of control plants (Figure 4a,b). They had normally formed upper and lower epidermis, palisade and spongy parenchyma and vascular bundle surrounded by parenchyma sheath (Figure 4a-b). Only difference could be observed in the smaller number of chloroplasts in spongy parenchyma cells of leaves from media with 0.1 g Pb·l^−1^ (Figure 4e). More pronounced differences were noted in the chloroplast ultrastructure (Figure 4c–h). Chloroplast of plants exposed to higher concentration of Pb (0.1 g·l^−1^) had swollen stroma, dilatated or poorly formed grana thylakoids, and more plastoglobules (Figure 4f–h) in comparison to chloroplasts of control plants and plants exposed to lower Pb concentration (0.05 g·l^−1^) (Figure 4b–d). We did not observe differences in ultrastructure between chloroplasts from control and 0.05 g Pb·l^−1^ plants (Figure 4c,d).

#### 2.5.5. Antioxidant Enzymes Protection

**Figure 5:**
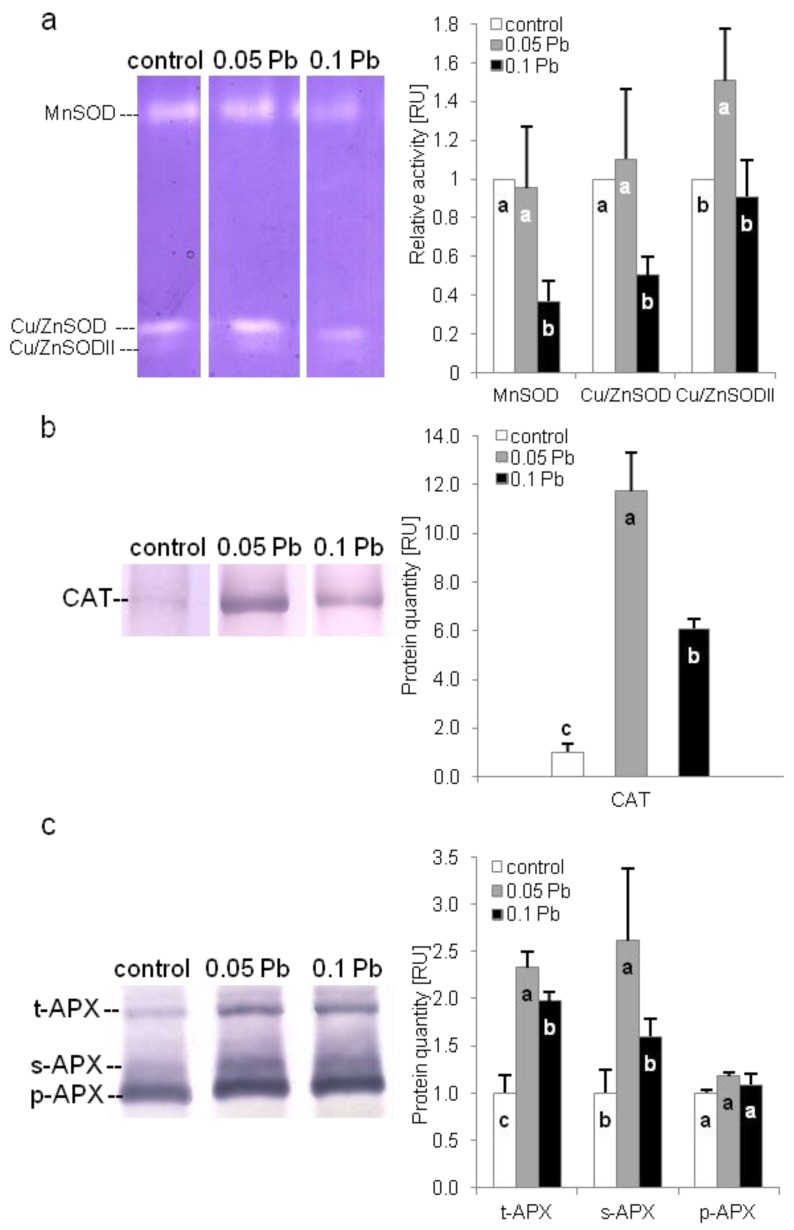
Antioxidant enzyme activity or content of Ceylon leadwort (*P. zeylanica*) shoots after 4 weeks of culture on the media with different Pb concentration; (**a**) activity of different superoxide dismutase (SOD) isoforms; (**b**) catalase (CAT) content; (**c**) content of thylakoid (t), stromal (s) and peroxisomal (p) ascorbate peroxidise (APX) (different letters—statistically significant difference within each parameter at *p* ≤ 0.05; RU-relative units).

The activity of three superoxide dismutase (SOD) isoforms, namely MnSOD, Cu/ZnSOD, and Cu/ZnSODII, were evaluated in shoots of *P. zeylanica* plants using native polyacrylamide gel electroforesis (PAGE). Higher concentration of Pb significantly reduced activity of MnSOD and Cu/ZnSOD isoforms. While Cu/ZnSODII activity increased in *Plumbago* plants exposed to 0.05 g Pb∙l^−1^ (Figure 5a). Catalase (CAT) and ascorbate peroxidase (APX) isoforms content were determined by SDS-PAGE electrophoresis and immunoblotting. Catalase accumulation increased in *P. zeylanica* shoots under Pb stress (Figure 5b). Higher accumulation was observed on medium with 0.05 g Pb∙l^−1^. Level of three APX isoforms: thylakoid (t-apx), stromal (s-apx) and peroxisomal (p-apx) were estimated in *P. zeylanica* shoots (Figure 5c). Thylakoid isoform of APX increased in plants cultivated on media with Pb. However, higher t-apx accumulation were recorded on media with lower Pb concentration. In turn, stromal isoform increased only in plants cultivated on medium with 0.05 g Pb∙l^−1^. Meanwhile, peroxisomal isoform accumulation did not change under Pb stress.

## 3. Discussion

Lead ions enter plants mainly through roots. The first and constitutive protecting mechanism of plants against lead toxicity is absorbing Pb ions on the root surface, thus limiting their penetration inside the plant [31]. After entering the root, lead ions can move apoplastically [32]. However, due to their high affinity for carboxylic groups, they are bound to cell wall components and retained there [33]. Nevertheless, this barrier is insufficient with higher concentrations of lead and prolonged stress [34]. When lead ions get inside the cells, the plant starts the next stage of defense relying on binding Pb ions with organic ligands (organic acids, amino acids and thiols) and/or sequestration in vacuoles [32]. This immobilization of lead in the roots allows the plant to protect aerial parts, especially organs and cells where the key process for plant life, i.e., photosynthesis, takes place [35]. Moreover, few plants belong to the group of hyperaccumulators that are capable of transporting large amounts of lead to shoots. However, in most plants, transport of small Pb amounts to shoots is observed [36]. In presented studies, the way of lead accumulation in Ceylon leadwort, depending on lead concentration in medium, was examined for the first time. The results indicate that, as in most plants, lead in large quantities is immobilized in the root system. However, increasing lead concentration in the medium twice (from 0.05 to 0.1 g∙l^−1^) caused over three times more Pb ions to be transported to aerial parts. The high toxicity of lead is related to the fact that even a small amount of ions getting into the symplast has an adverse effect on plants, interacting with cellular components and disrupting physiological processes [13,36,37].

The easiest to observe, nonspecific symptoms of lead toxicity are plant growth and development disorders. In many plants exposed to Pb ions, the first visible stage of these disorders is rapid reduction of root growth [8,20]. Pb ions, binding to the components of the cell wall, lead to its mineralization and loss of plasticity. In addition, Pb disrupts the microtubule network in the cell. All this leads to disorders in cell division, cell elongation and morphogenesis processes [32]. Similarly, in the case of the species studied, morphogenetic processes (root formation) were clearly disturbed on the medium with a higher concentration of lead. In turn, cell division and the process of cell elongation, and thus root growth, were more susceptible to lead ions as they were also interfered by the lower Pb concentration. Limitation of plant growth as a result of lead toxicity (disturbance of water and mineral management) is also manifested in the decrease of both fresh and dry weight of shoots and roots [8,13]. In *P. zeylanica* plants, we observed not only no decrease in dry weight of the organs, but also an increase in shoot dry weight. This may indicate some tolerance of this species to lead despite limitations in root growth.

No change in the content of malondialdehyde (MDA) in root tissues may point to some level of *P. zeylanica* tolerance to lead. Malondialdehyde, as a product of the decomposition of the lipid hydroperoxide unstable molecules, is evidence of the lipid peroxidation process [38]. Lipid peroxidation is a result of activity of reactive oxygen species (ROS), generated by the action of lead ions. Lipid peroxidation leads to changes in the structure of cell membranes increasing their permeability and damaging the membrane proteins [11,38]. Lack of increase in MDA content in *Plumbago* shoot tissues under lower lead ions concentration may indicate a well-functioning antioxidant system scavenging ROS. However, the antioxidant system in shoot cells was not sufficient to cope with higher lead concentration in the medium which was reflected in the increase of MDA. This corresponds with results obtained for activity of antioxidant enzymes and contents of phenols which were higher in shoots from 0.05 g Pb·l^−1^ than in shoots from 0.1 g Pb·l^−1^ treatment.

Pb toxicity in *P. zeylanica* shoots were manifested in the decreased content of photosynthetic pigments, both total chlorophylls and carotenoids. A reduction of the pigments content in the presence of lead ions is related to both their degradation and synthesis inhibition [8,13]. Chlorophyll degradation is due to lead-induced chlorophyllase activity [14]. However, inhibition of pigment synthesis has a greater importance. Lead can inhibit chlorophyll synthesis directly by disturbed synthesis of its precursor—porphobilinogen by lowered ALAD activity resulting from inactivation of enzyme active side (lead affinity to—SH groups) [14] and indirectly, as a result of impaired mineral metabolism (lack of necessary ions, especially Mg^2+^) [39]. Pb inactivates also enzymes associated with carotenoid synthesis pathway (MEP-methyl eritrythol phosphate pathway) [40]. It is believed that more sensitive to lead ions is chlorophyll *b* [41]. Our research does not confirm this, because the ratio of Chl *a*/*b* decreased significantly under the influence of a higher concentration of lead in the medium.

A decrease in the content of carotenoids and chlorophylls, especially Chl *a*, is one of the factors leading to changes in the chloroplast ultrastructure of plant growing in conditions of high lead concentration [37]. The reason for this is that the formation and stabilization of chloroplast grana depend on the LHCII complex, half of which are chlorophyll molecules [42]. Further, a reduction of protein Lhcb1 complex content may indicate changes in chloroplast structure. The level of changes in the chloroplast ultrastructure is linked with Pb concentration [37]. Low Pb doses make no or small ultrastructure disturbances [20,43,44] but high ones lead to serious chloroplast remodeling like swelling or shrinkage of whole organelle and loss and relaxation of thylakoid grana [45,46,47,48]. Changes in chloroplast structure result from the affinity of lead for amino and thiol groups of thylakoid membrane structural proteins [49]. Similarly as in other research, *P. zeylanica* chloroplasts from leaves of plants treated with lower lead concentration (0.05 g Pb∙l^−1^) had no changed ultrastructure, but treated with higher one showed some swelling and relaxation of thylakoid grana, which was in accordance with the observed decrease in the content of chlorophylls and protein Lhcb1 complex. On the other hand we observed increase of RuBisCo content which as a large and spatial stromal protein partisipates in maintenance of forming grana [50,51]. This may indicate some acclimatization mechanisms activated by *Plumbago* plants. The evidence of acclimatization process in *P. zeylanica* shoots are also plastoglobules observed mainly in chloroplasts from 0.1 g Pb∙l^−1^ medium but also sporadically in chloroplasts from 0.05 g Pb∙l^−1^ (Figure 4d,h). Plastoglobules are believed to be reservoirs and synthesis sites of neutral lipids which are used to rebuild membrane lipids during stress [42], and thus can stabilize the photosynthetic apparatus [20]. Otherwise, changes in the arrangements of granal and stromal thylakoids of chloroplasts affect directly both linear and cyclical electron transport between photosystems [34,48].

Lead causes detrimental effects on light-dependent and light-independent reactions of photosynthesis [37]. In Photosystem II (PSII), the first place of light reactions, it influences donor side (water splitting complex), light harvesting complex (LHC), reaction center (RC) and acceptor side (plastoquinone) [39,52]. In *Plumbago* shoots we observed that high lead concentration negatively affected water splitting (oxygen evolving—OEC) complex. The main role in this complex is played by the PsbO protein, one of the extrinsic subunits of PSII, also called the water splitting enzyme of photosynthesis, which stabilizes Mn cluster (Mn_4_CaO_5_) and optimizes the levels of cofactors (Ca^2+^ and Cl^-^) necessary for the water splitting process [53]. OEC can be damaged by substitution of Mn^2+^ and/or Ca^2+^ ions with Pb^2+^ ions and by conformational changes arising from Pb association with protein thiol groups [54]. In *Plumbago* shoots from medium with 0.1 g Pb∙l^−1^, lead ions caused significant decrease of water splitting complex activity (F_V_/F_0_) despite an increased PsbO protein quantity. This led to a limitation in electron flow on the donor side of PS II RC.

The amount of energy transferred to the RC PSII depends on the size and efficiency of the main LHC II external antennas, which include Chl *a* and mostly Chl *b* [20,37,51]. By inhibiting the synthesis of chlorophylls and activation of chlorophyllase, Pb causes a size decrease of the LHC II. Moreover, Pb ions may cause monomerization of PSII light harvesting complex proteins and inhibit synthesis of the light harvesting core proteins [55,56]. In *Plumbago* shoots from high lead concentration, the reduced amount of Lhcb1 protein, one of three proteins building LHCII, in conjunction with chlorophyll *b* content decrease (data not shown) indicates disturbances in the amount of energy transferred to the RC of PSII.

In the PSII reaction center, Pb interferes mainly with Chl *a* molecules, impairing their function by the substitution of Mg^2+^ ions in porphyrin rings [57], leading to their constant excitation, and thus permanent inactivation of RC [58]. As we mentioned earlier, content of chlorophylls decreased only in *Plumbago* shoots treated with the high lead concentration. Besides Chl molecules, Pb attacks structural proteins of RC, like D1 (PsbA) and D2 protein, by association with their—SH groups and thus changing their spatial structure, obstructing transport of electrons outside PSII [59]. In our research, the increase of PsbA protein content in *Plumbago* shoots cultivated on medium with 0.1 g Pb∙l^−1^, indicates its rapid damage and attempts to its quick reconstruction. Damage of the PSII reaction centers is also confirmed by a decrease of Vj, which indicates the number of active reaction centers to all reaction centers that can be closed [60]. This is also reflected in the decrease in the number of active reaction centers per cross section (RC/CS_0_). In PsbA protein, lead often associates with Q_B_ site preventing electron transport between Q_A_ and Q_B_ sites but also beyond Q_B_ [30,61]. In case of *P. zeylanica* shoots, we noted decrease of φ_Po_ and increase of ψ_Eo_, what informs on the one hand about reduced electron flow efficiency between Q_A_ and Q_B_ and on the other hand about the increase of probability that trapped exciton moves an electron beyond Q_B_ in these plants. Undisturbed electron transport rate beyond Q_B_ could come out of larger plastoquinone pool (Area) resulting in higher amount of total electron carriers per RC (Sm). Furthermore, this points out that PSII acceptor side of Pb-treated *Plumbago* shoots were not affected by lead action, which can be observed in other plants [52]. Similar plant response to Pb toxicity was noted in kidney vetch plants [19]. 

Photosystem I (PSI) appears to be relatively resistant to lead ions. However, lead-induced changes in thylakoid membrane properties affect electron transporters, like plastocyanin (PC) and ferredoxin (Fd) [55]. Moreover, Pb can also directly impacts these transporters. It reduces synthesis and activity of PC (substitution of copper ions) and limits activity of NADP+ ferredoxin reductase (combining with thiol groups) thus suppressing reduction power (NADPH) synthesis [62]. Disorders at this stage of photosynthesis could be manifested by decrease of maximal fluorescence (F_M_), Q_B_ oxidation (δ_Ro_), the efficiency of quantum yield for reduction of PSI end acceptors (φ_Ro_) and the efficiency of electron transport (ρ_Ro_) [30,63]. In the case of *P. zeylanica* plants growing under high lead stress, we observed on the one hand a significant reduction of F_M_ but on the other a significant increase of δ_Ro_, φ_Ro_, and ρ_Ro_. Moreover, a decrease of V_I_ value suggests that the efficiency of electron transport was not disturbed especially from Q_B_ to the PSI end electron acceptors. Decrease of fluorescence can be explained by the reduced chlorophyll content [28]. Whereas, increase of other parameters may be result of changing plastoquinone pool but also the effective cycle electron flow (CEF) or/and other alternative electron cycles.

Some authors believe that light-independent reactions of photosynthesis are more susceptible to Pb, because of Pb-induced interferences in synthesis and activity of Calvin-Benson cycle enzymes, mainly RuBisCo [9,15,64]. This point of view is also indirectly confirmed in our research. Although, RuBisCo content not only did not decrease, but increased in *Plumbago* shoots cultivated under both tested Pb concentrations. Because RuBisCo is abundant in plant cells, increased accumulation points to the problems with its efficiency [65].

Under natural conditions, the photosynthetic apparatus may be exposed to the potentially damaging effects of excessive radiation [66,67]. Under conditions of abiotic stress, including heavy metals, which limit photosynthetic metabolism, even moderate radiation may be excessive and activating dangerous photo-oxidative reactions, which lead to emerging highly reactive singlet oxygen [37,68,69]. The primary defense mechanism of plants is based on the harmless emission of absorbed energy as heat and is called non-photochemical energy dissipation/conversion [68]. Parameters such as Y(NPQ), Y(NO), and Y(II) allow to describe the excitation energy fate and show how plants cope with excess excitation energy and indirectly with arising stress [70]. The quantum photosynthetic yield of PSII (Y(II)) is the energy photo-chemically converted in PSII. Y(NPQ) and Y(NO) correspond to non-photochemical quenching, thus energy dissipated in the form of a heat or fluorescence. The first one reflects regulated energy dissipation via the photoprotective NPQ-mechanism. The second one corresponds to passive energy dissipation through non-photo-protective mechanisms [70]. Plants exhibit a high photoprotective capacity when values of Y(II) are maximal and Y(NPQ) are higher than Y(NO). On the other hand, eventually photodamage can be reflected by high values of Y(NO) and low values of Y(NPQ) [70]. In the case of our study, we noted statistically higher value of Y(II), lower value of Y(NO) and no change in value of Y(NPQ) in plants from 0.05 g Pb∙l^−1^ in comparison to control plants what means that photoprotective mechanisms worked flawlessly. In contrast, in plants from 0.1 g Pb∙l^−1^, we recorded no changes in values of Y(NPQ) and Y(NO) what can mean that NPQ-generated reactions were not affected by emerging stress [70]. However, the quantum photosynthetic yield of PSII (Y(II)) in these plants were statistically lower what can correspond with diminish linear electron flow [71].

To trigger NPQ-mechanisms (xanthophyll cycle), it is necessary to create a proton gradient across the thylakoid membrane [71]. In the case of diminish linear electron flow, proton gradient may be generated via cycling electron flow. In stress conditions, plants switch over to cycling electron flow (CEF), which helps to dissipate excess energy and protect PSII from photoinhibition [72]. Taking into account Y(NPQ) value maintained at the control level and high level of energy dissipation (increased value of DI_o_/RC) in *Plumbago* leaves from medium with 0.1 g Pb∙l^−1^, we assumed that in these plants the electrons are redirected to cyclical transport around PSI to protect PSII.

Plants can preserve their photosystems not only by redirecting electrons to cycling flow but also via several other alternative electron transport pathways, like ‘water-water cycle’ (O_2_ reduction at the acceptor side of PSI followed by ascorbate peroxidase reaction) [73] and chlororespiration (terminal plastoquinone oxidase (PTOX)-mediated reduction of O_2_ when PQ-pool is over-reduced) [65,74]. The activation of these pathways is often observed in plants under stress conditions and connected with redox state unbalance influencing both ferrodoxin and PQ pool [75]. The impairment of redox state can result from the excessive energy absorbed and failure in its exploitation on the PSI acceptor side and/or in the dark reactions of photosynthesis. Rodriguez et al. [15] demonstrate that inactivation of ferredoxin and thioredoxin as well as Calvin–Benson cycle enzymes is the first effect of Pb ions toxicity on the photosynthetic apparatus. The malfunction of electron carriers and enzymes leads, in the first place, to inactivation of PSI, then to PQ pool overreduction and, consequently, to PSII photoxidation. To secure the efficient photosystems functioning, plants increase the activity of PTOX which catalyzes the electron transfer from PQH_2_ to O_2_ [65]. Under conditions of limitation on the acceptor side of the PSI, redundant electrons are redirected by PTOX to O_2_, thus allowing free flow of electrons beyond Q_B_ and preventing PSII photoxidation [62]. Inactivation of Calvin–Benson cycle enzymes lead to reduction of PQ pool by redirection of electron via two pathways: (1) ferredoxin (Fd)—ferredoxin-plastoquinone oxidoreductase (FQR)—nicotinamide adenine dinucleotide phosphate (NADPH); (2) Fd—chloroplastic NAD(P)H dehydrogenase complex (NDH). In the second pathway, the lumen is acidified, and NPQ mechanisms are activated [65,76]. It appears that the chlororespiration mechanism has been activated to secure efficient photosynthesis in *Plumbago* shoots from 0.05 g Pb∙l^−1^ medium. This is demonstrated on the one hand by efficient electron transport maintained beyond of Q_B_ (high ETR) and on the other by inefficient RuBisCo (as mentioned above) and slightly reduced values of the parameters φRo, δRo; ρRo indicating the limitation on the PSI acceptor side. In addition, a significant increase in the activity of Cu/Zn SODII and the amounts of CAT and stromal APX may suggest the generation of reactive oxygen species during the oxidation of reduced PQ by PTOX.

## 4. Materials and Methods

### 4.1. Plant Material

Experimental material comprised established in vitro culture of *Plumbago zeylanica* Linn. plants (from the collection of Department of Biotechnology and Horticulture, University of Agriculture in Krakow) growing on basal medium containing ½ MS [77] macro-, microelements and vitamins, with 30 g·l^−1^ of sucrose, solidified with 8 g·l^−1^ of agar, with no growth regulators and pH = 5.8 (prior autoclaving). Plant materials were cultured at 25 ± 1 °C under a 16/8 h light photoperiod of 50 μmol m^−2^ s^−1^ photosynthetic photon flux density.

### 4.2. Experimental Conditions and Stress Treatment

Explants (shoot fragments with two leaves) were obtained from initial in vitro cultures and placed on basal medium with addition of lead nitrate (Pb(NO_3_)_2_) at concentrations: 0.0 (control), 0.05, and 0.1 g Pb∙l^−1^. In vitro cultures were kept at the same temperature and light conditions as initial plant material. For each experimental treatment, five explants each were placed in ten vessels. The whole experiment was repeated in triplicate.

### 4.3. Biometric Assay

After 4 weeks of culture, the growth index (GI) of plants was estimated. Growth index was determined according to formula: GI[%] = (FFW − IFW)/FFW∗100, where FFW is final fresh weight of shoot and IFW is initial fresh weight of shoot. Rooting efficiency was estimated as rooting percentage and average length of the longest root per explants. Shoots and roots were separately freeze-dried for 48 h and percentage of dry weight content were counted.

### 4.4. Estimation of Lead Concentration in Plant Tissue

Plant freeze-dried samples (200 mg) were digested in 5 mL HNO_3_ and 1.65 mL H_2_O_2_ to dissolve all elements presented in plant tissue. Samples were filtered, adjusted to 25 mL with MQ-water and analyzed with Atomic Absorption Spectrometry (flame atomic absorption spectroscopy [FAAS] or graphite furnace atomic absorption spectroscopy [GF-AAS], equipped with Zeeman effect background correction and an CSX 260 auto-sampler [Thermo Scientific, Waltham, MA USA]), to determine the metal concentrations. Results have been expressed as mg Pb∙kg^−1^ of DW.

### 4.5. Estimation of Lipid Membranes Peroxidation Level

To estimate peroxidation level of lipid membranes, malondialdehyde (MDA) content was measured, separately in shoots and roots, according to Dhindsa et al. [78] with modifications. In brief, plant material (ap. 10 mg of DW) were homogenized in 1.0 mL of 0.1% TCA (trichloroacetic acid) and centrifuged (4 °C, 15 min, 10,000× *g*). The, 0.2 mL of supernatant was collected and mixed with 0.8 mL of 20% TCA containing 0.5% of TBA (thiobarbituric acid). Then, samples were incubated 30 min in 95 °C, after that cooled on ice, and centrifuged (4 °C, 10 min, 10,000× *g*). The absorbance of mixtures was measured at 532 nm and 600 nm, using Double Beam spectrophotometer U-2900. The value at 532 nm (A_532_) was reduced by the value at 600 nm (A_600_, the correction value): A_x_ = A_532_ − A_600_. The concentration of MDA was calculated using absorbance coefficient for MDA ε = 155 mM^−1^cm^−1^, and expressed as nM MDA per 1 g of DW.

### 4.6. Phenolic Compounds’ Content Determination

#### 4.6.1. Total Phenols

To estimate content of total phenolic compounds in shoots and roots, photometric method by Swain and Hillis [79] with Folin–Ciocalteu (F-C) reagent was used, with small modifications. Dry plant tissue (10 mg) was extracted in 1 mL of 80% methanol and centrifuged (4 °C, 15 min, 20,000× *g*). 1 mL of diluted extract was mixed with 0.2 mL of F-C reagent (Sigma-Aldrich Chemie, GmBH, Steinheim, Germany) and 1.6 mL of 5% Na_2_CO_3_. Reaction mixture was incubated 20 min in 40 °C. After incubation, the absorption of samples was measured at 740 nm, using double beam spectrophotometer U-2900. Chlorogenic acid was used as standard. The content of phenolic compounds was expressed as milligram of chlorogenic acid per 1 g of DW.

#### 4.6.2. Specific Phenol Groups

The content of cinnamic acid derivatives (CAD), flavonols, and anthocyanins was determined using the spectrophotometric method according to Fukumoto and Mazza [80]. Plant material (10 mg) was homogenized in 80% methanol in 4 °C. Samples were centrifuged for 15 min (20,000× *g*, 4 °C). Diluted supernatant was mixed with: 0.25 mL 0.1% HCl in 96% EtOH and 4.55 mL 2% HCl in H_2_O. After 15 min, the absorbance was measured at wavelengths of 320, 360, and 520 nm. Using calibration curves made for caffeic acid, quercetin, and cyanidin, the content of CAD, flavonols, and anthocyanins was calculated. Results were expressed as milligram of CAD, flavonols, and anthocyanins per 1 g of DW.

#### 4.6.3. Plumbagin

Determination of plumbagin concentration in shoot and roots of *P. zeylanica* plants have been performed using high-pressure liquid chromatography (HPLC). Briefly, 10 mg of dry plant tissue was extracted in 0.5 mL MQ-water and 0.5 of tetrahydrofuran (THF) and sonicated per 30 min. Next, 200 mg of NaCl was added. Samples were shaken to dissolve the salt and centrifuged (4 °C, 15 min, 20,000× *g*). The supernatant was used in HPLC analysis. The chromatographic separation was carried out using Beckmann Gold System (Beckman Coulter, Brea, CA, USA) equipped with a Thermo Separations Spectra 100 variable wavelength detector (Thermo Scientific, Waltham, MA, USA) and a Rheodyne 6-way injection valve. For the stationary phase, an Agilent XDB-C18 (4.6 × 50 mm, 1.8 µm) was used. The flow rate used was 1 mL∙min^−1^. The sample injection volume was 10 µL. The mobile phase for the analysis consisted of methanol as eluent A and water as eluent B. The separation was performed in isocratic conditions (60% A). The length of the analysis was 5 min. The retention time of plumbagin was 2.78 min. The separation was carried at room temperature. For determining the concentration of plumbagin a 4-point, 3-level standard curve was used. Monitoring was performed at 254 nm.

### 4.7. Evaluation of Photosynthetic Apparatus Efficiency

#### 4.7.1. Photosynthetic Pigment Content Estimation

To estimate photosynthetic pigment content photometric method by Lichtenthaler [81] with modifications has been used. Briefly, dry shoot tissue (10 mg) was extracted in 1 mL of 80% acetone with magnesium chloride, in ice-cold conditions. Samples were centrifuged (4 °C, 15 min, 20,000× *g*) and supernatant were collected in glass cylinder, while samples were extracted two more times with 1 mL of 80% acetone with magnesium chloride and centrifuged (4 °C, 15 min, 20,000× *g*). Supernatant was diluted to 5 mL with 80% acetone with magnesium chloride. Samples were mixed and the absorbance was measured at 663 nm (Chl *a*), 646 nm (Chl *b*) and 470 nm (Car), using double beam spectrophotometer U-2900 (Hitachi High-Technologies Corporation, Tokyo, Japan). Content of pigment was calculated during Wellburn [82].

#### 4.7.2. Chlorophyll a Fluorescence Measurement

Chlorophyll *a* fluorescence induction curves analysis were done on in vitro plants’ leaves. Leaves from 15 plants of each combination were adapted to the dark for 25 min. Fast kinetics chlorophyll fluorescence induction were measured using Handy-PEA (Hansatech, King’s Lynn, UK) spectrofluorometer on the basis of the relevant standard procedures. The fluorescence were induced by red light: λmax = 650 nm, 2000 μmol∙m^−2^∙s^−1^. Selected functional and structural photosynthetic parameters (Table 3) were calculated ac. to Jiang et al. [63] and Kalaji et al. [83]. Recorded curves were analysed using the fluorometer producer’s soft-ware (PEA-Plus). Slow kinetics chlorophyll fluorescence induction were measured using OS1p chlorophyll fluorometer (Opti-Sciences, Hudson, NH, USA). The fluorescence were measured using a modulated light source of 25 µmol∙m^−2^∙s^−1^ at 660 nm and a saturation pulse from a white light light-emitting diode with an intensity of 7700 µmol∙m^−2^∙s^−1^ for a duration of 0.8 s. Quantum photosynthetic yield of PSII (Y(II)), photoprotective non-photochemical quenching (Y(NPQ)) and all other non-photo-protective non-photochemical quenching Y(NO) were evaluated using quenching tests protocol and electron transport rate (ETR) using rapid light curves (RLC) protocol.

#### 4.7.3. Protein Content Determination

To determine the content of proteins: ascorbate peroxidase (APX), catalase (CAT), rubisco large subunit (RbcL) and photosystem’s proteins, like: PsbA, Lhcb1 and PsbO plant tissue (shoots) were extracted in buffer according to Laureau et al. [84] with modifications, in 4 °C. Protein concentration was determined according to Bradford [85] with BSA as a standard. SDS-PAGE were performed at 4 °C, with 24 mA for 15 min, 34 mA for 35 min and 68 mA for 60 min, on 12% polyacrylamide gels. For each line 5 µg of protein extract was applied. Blotting was performed on a polyvinylidene fluoride membrane (PVPD) (BioRad). Identification of protein bands was done with polyclonal antibodies (Agrisera), and detection was performed with alkaline phosphate buffer with BCIP/NBT as a substrate. Membranes were scanned, and the intensity of bands was calculated using ImageJ version 1.52n (open source software, OSS). The content of each protein was expressed as arbitral units, defined as area under the curve per µg of protein applied to the line. After analysis, the highest protein quantities at each gel were expressed as 1, and all data were normalized to it.

#### 4.7.4. Histological and TEM Observation

After 4 weeks of culture, fragments of leaves and roots were incubated in 2% paraformaldehyde and 2% glutaraldehyde in 0.1 M cacodylate buffer (pH 7.2) for 2 h, rinsed 4 times in cacodylate buffer, and post-fixed in a solution of 2% osmium tetroxide in cacodylate buffer for 3 h at 4 °C. Plant tissue was dehydrated through a graded ethanol series and substituted by propylene oxide, and then embedded in glycid ether 100 epoxy resin (SERVA) equivalent to the former Epon812. The resin was polymerized at 65 °C for 24h. Semi-thin sections were prepared with Jung RM 2065 microtome, stained with methylene blue and azur A, and examined under a light microscope (Olympus-Provis, Tokyo, Japan). Ultra-thin sections were prepared with Ultracut UCT Leica microtome, collected on formvar coated grids and stained with uranyl acetate followed by lead citrate for 1 min. Examinations were made under a transmission electron microscope (Morgagni 268D, Hillsboro, OR, USA).

#### 4.7.5. Evaluation of Antioxidant Enzymes

The activity of SOD isoforms was examined according to Miszalski et al. [86] with modifications. Briefly, native polyacrylamide gel electrophoresis (PAGE) with specific staining for SOD was used to separate and visualized SOD isoforms. For protein isolation 0.1 M phosphate buffer with DTT, EDTA and PVPP, with pH = 7.5 was used. Protein concentration was determined according to Bradford [85] with BSA as a standard. PAGE was performed at 4 °C, 180V, 1 h on 13% polyacrylamide gels, with electrophoresis buffer according to Laemmli [87] with no SDS addition. For each line, 20 µg of protein extract was applied. Activity of SOD isoforms was visualized after 30 min incubation of gels in the darkness at the room temperature, after exposition to daily light. Incubation was performed in staining buffer described by Miszalski et al. [86]. Gels were scanned, and intensity of bands were calculated using ImageJ (open source software, OSS). Activity of isoforms was expressed as arbitral units, defined as area under the curve per µg of protein applied to the line. After analysis, the highest protein quantities at each gel were expressed as 1 and all data were normalized to it.

### 4.8. Statistical Analyses

All biometric parameters and chl *a* fluorescence measurements were done in 10 replicates, all spectrophotometric analyses were done in 5 replicates and AAS, HPLC and electrophoresis analyses were done in 3 replicates. All results were subjected to one-way analysis of variance (ANOVA). The significant differences between means were determined with DUNCUN test at *p* < 0.05 level. Statistica 12.0 (StatSoft Inc., Tulsa, OK, USA) was used to carry out statistical analyses. Results obtained for the shoots and roots in vitro plants were separately statistically verified.

## 5. Conclusions

The photosynthetic apparatus of *Plumbago* plants cultivated on media with lower Pb concentration was characterized by no changes in both the content of photosynthetic pigments and the size of LHC II antennas, as well as the lack of limitation on the RC PSII donor side. As a result, no changes in the efficiency and performance of the PSII compared to the control plants were observed. At the same time, in these plants statistically significantly higher activity of Cu/Zn-SODII chloroplast isoform and content of stromal ascorbate peroxidase and catalase were noted, indicating a high risk of limitation on the acceptor side of PSI. It seems that in these plants the mechanism of acclimatization of the photosynthetic apparatus to elevated Pb concentration in shoots is associated with the decoupling of linear electron transport in favor of alternative electron flows, i.e., pseudocyclic (chlororespiration). Such a mechanism would allow for the maintenance of a stable oxidoreductive balance of chloroplasts and no risk of PSI photooxidation.

In plants cultivated on a medium containing higher Pb concentration, a significant decrease in the photosynthetic pigments content, the size of LHC II photosynthetic antennas, the amount of active RC PSII, and OEC activity were observed. Moreover, a significant remodeling of the chloroplast ultrastructure, decreasing the number of grana thylakoids in favor of stromal thylakoids appeared. These results indicate a significant limitation of PS II on the donor side and reaction center resulting in a PSII performance decrease. However, the lack of limitation on the PSI acceptor side may suggest that the acclimatization mechanism of *Plumbago* plants to higher Pb concentration is associated with the dominance of cyclic electron transport over linear transport. This strategy would allow plants the most effective use of a limited amount of absorbed light energy while reducing the risk of both PSII and PSI photoxidation.

Presented research indicates that *P. zeylanica* plants accumulate large amounts of lead in roots, which is very important considering that this organ is mainly used for preparation of pharmacologically important extracts [88]. Furthermore, *P. zeylanica* acclimates to lead toxicity by adjusting photosynthetic apparatus via different mechanisms depending on Pb ion concentration.

## Figures and Tables

**Table 1:** Growing parameters of Ceylon leadwort (*P. zeylanica*) plants depending on Pb concentration in the medium after 4 weeks of culture (*different letters*—statistically significant difference within each parameter at *p* ≤ 0.05).

Pb Concentration [g/l]	Parameters [±SD]				
	Growth Index [%]	Shoot Dry Weight Content [%]	Rooting [%]	Root Length [cm]	Root Dry Weight Content [%]
0.0 (control)	69.0*a* ±12.2	11.1*b* ±1.1	92.0*a* ±9.8	6.3*a* ±1.6	12.7*a* ±1.7
0.05	73.5*a* ±10.3	11.2*b* ±0.9	96.0*a* ±8.9	5.0*b* ±1.0	13.3*a* ±1.2
0.1	71.8*a* ±13.9	12.4*a* ±0.9	74.3*b* ±19.0	4.3*c* ±1.3	12.2*a* ±1.3

**Table 2:** Lipid peroxidation level and phenolic compounds content in shoots and roots of Ceylon leadwort (*P. zeylanica*) plants depending on Pb concentration in the medium after 4 weeks of culture (*different letters*—statistically significant difference within each parameter and organ at *p* ≤ 0.05).

Pb Concentration [g/l]	Organ	Parameters [±SD]					
		MDA* [nM∙g^−1^DW]	TP [mg∙g^−1^DW]	CAD [mg∙g^−1^DW]	FLAV [mg∙g^−1^DW]	ANT [mg∙g^−1^DW]	PLUMB [mg∙g^−1^DW]
**0.0 (control)**	shoot	180.9*b* ± 2.4	44.6*a* ± 0.7	12.0*a* ± 0.7	11.0*a* ± 0.7	4.4*b* ± 0.4	33.3*a* ± 3.5
0.05		179.8*b* ± 5.1	41.6*b* ± 0.7	12.4*a* ± 0.5	11.5*a* ± 0.5	3.0*a* ± 0.2	30.4*a*± 0.5
0.1		185.7*a* ± 2.8	39.5*c* ± 2.0	11.2*a* ± 1.3	9.6*b* ± 1.2	1.7*c* ± 0.4	28.5*a* ± 1.0
0.0 (control)	root	261.3*a*± 16.3	27.8*b* ± 1.5	5.1*a* ± 0.2	2.7*a* ± 0.1	1.0*a* ± 0.1	14.7*a* ± 1.0
0.05		236.7*a*± 11.5	37.5*a* ± 0.8	4.3*b* ± 0.2	2.3*b* ± 0.2	0.7*b* ± 0.1	13.2*b* ± 0.8
0.1		251.3*a*± 5.9	43.9*a* ± 8.1	3.7*c* ± 0.3	2.2*b* ± 0.2	0.7*b* ± 0.1	12.1*b* ± 0.0

* MDA—malondialdehyde; TP—total phenols; CAD—cinnamic acid derivatives; FLAV—flavonoles; ANT—anthocyanins; PLUMB—plumbagine.

**Table 3:** Extracted and calculated parameters of fast kinetics Chl *a* fluorescence (according to Jiang et al. [63], Kalaji et al. [83]).

**Extracted parameters**		**Calculated parameters**	
F_O_	Minimum fluorescence, when all PSII reaction centers (RCs) are open	Fv	variable fluorescence; Fm − F_0_
F_M_	Maximum fluorescence, when all PSII reaction centers are closed	Fv/Fm	maximum quantum yield of PSII; (Fm − F_0_)/Fm
F_50μs_, F_100μs_, F_300μs_, F_2ms_, F_30 ms_	Fluorescence intensities at 50, 100, 300 μs, 2, 30 ms, respectively	Fv/F_0_	activity of the water-splitting complex on the donor side of the PSII; (Fm − F_0_)/F_0_
Area	Total complementary area between fluorescence induction curve and *F* = *F_m_*		
**OJIP parameters (calculated)**		**Yields or flux ratios (calculated)**	
V_J_	Relative variable fluorescence at 2 ms (J-step); *V_J_* = (*F_2ms_* − *Fo*)/(*Fm* − *Fo*)	φ_Po_	Maximum quantum yield of primary photochemistry at t = 0; *φ_Po_* = *1* − *Fo*/*Fm* = *Fv*/*Fm*
V_I_	Relative variable fluorescence at 30 ms (I-step); *V_I_* = (*F_30ms_* − *Fo*)/(*Fm* − *Fo*)	φ_Eo_	Quantum yield for electron transport at t = 0; *φ_Eo_* = (*Fv*/*Fm*)(*1* − *V_J_*)
S_m_	Normalized total complementary area above the OJIP transient (reflecting multiple-turnover *Q_A_* reduction events) or total electron carriers r RC; *S_m_* = *Area*/(*Fm* − *Fo*)	ψ_Eo_	Probability (at time 0) that trapped exciton moves an electron into the electron transport chain beyond; *ψ_Eo_* = *1* − *V_J_*
		ρ_Ro_	Efficiency with which a trapped exciton can move an electron into the electron transport chain from *Q_A‾_* to the PSI and electron acceptors; *ρ_Ro_* = *ψ_Eo_δ_Ro_* = (*1* − *V_J_*)(*1* − *V_I_*)/(*1* − *V_J_*)
		δ_Ro_	Efficiency with which an electron can move from the reduced intersystem electron acceptors to the PSI end electron acceptors; *δ_Ro_* = *RE_o_*/*ET_o_* = (*1* − *V_I_*)/(*1* − *V_J_*)
		φ_Ro_	Quantum yield for the reduction of end acceptors of PSI per photon absorbed; *φ_Ro_* = *RE_o_*/*ABS* = *φ_Po_ψ_Eo_δ_Ro_*
**Specific fluxes or activities per reaction center (RC) (calculated)**		**Phenomenological fluxes or activities per excited cross section (CS) (calculated)**	
ABS/RC	Absorption flux per RC; *ABS*/*RC* = *Mo*/*V_J_* = *4*(*F_300μs_* − *Fo*)/(*Fm* − *Fo*)/*V_J_*	TR_o_/CS_o_	Trapped energy flux per CS at t = 0; *TR_o_*/*CS_o_* = (*ABS*/*CS_o_*)φ_Po_
TR_o_/RC	Trapped energy flux per RC at t = 0; *TR_o_*/*RC* = *Mo*/*V_J_*	ET_o_/CS_o_	Electron transport flux per CS at t = 0; *ET_o_*/*CS_o_* = (*ABS*/*CS_o_*)*φ_Eo_*
ET_o_/RC	Electron transport flux per RC at t = 0; *ET_o_*/*RC* = (*Mo*/*V_J_*)*ψ_Eo_*	DI_o_/CS_o_	Dissipated energy flux per CS at t = 0; *DI_o_*/*CS_o_* = *ABS CS_o_* − *TR_o_*/*CS_o_*
DI_o_/RC	Dissipated energy flux per RC at t = 0; *DI_o_*/*RC* = *ABS*/*RC* − *TR_o_*/*RC*		
**Density of reaction centers (calculated)**			
RC/CS_o_	Amount of active PSII RCs per CS at t = 0; *RC*/*CS_o_* = *φ_Po_*(*ABS*/*CS_o_*)(*V_J_*/*Mo*)		

## Abbreviations

ALAD	alpha amino levulinic dehydrogenase
APX	ascorbate peroxidase
BCIP	5-bromo-4-chloro-3-indolyl phosphate
BSA	bovine serum albumin
CAD	cinnamic acid derivatives
CAT	catalase
CEF	cyclic electron flow
CS	cross section
Cu/ZnSOD	copper- and zinc-containing superoxide dismutase
Cu/ZnSODII	chloroplastic copper- and zinc-containing superoxide dismutase
DTT	dithiothreitol
DW	dry weight
EDTA	ethylenediaminetetraacetic acid
ETR	electron transport rate
F-C	Folin–Ciocalteu
Fd	ferredoxin
FFW	ferredoxin
FQR	ferredoxin-plastoquinone oxidoreductase
GI	growth index
HPLC	high performance liquid chromatography
IFW	initial fresh weight
Lhcb1	light-harvesting chlorophyll a/b binding protein
LHCII	light-harvesting complex of PSII
MDA	malondialdehyde
MEP	methyl eritrythol phosphate pathway
MnSOD	manganese superoxide dismutase
MQ	Milli-Q
MS	Murashige and Skoog medium
NADP+	nicotinamide adenine dinucleotide phosphate
NADPH	reduced nicotinamide adenine dinucleotide phosphate
NBT	nitro blue tetrazolium
NDH	NAD(P)H dehydrogenase complex
NPQ	non-photochemical quenching
OEC	oxygen evolving complex
PC	plastocyanin
PQ	plastoquinone
PsbA	Photosystem II reaction center protein A
PsbO	oxygen-evolving enhancer protein 1
PSI	Photosystem I
PSII	Photosystem II
PTOX	terminal plastoquinone oxidase
PVPD	polyvinylidene fluoride membrane
PVPP	polyvinylpolypyrrolidone
RC	reaction center
RbcL	rubisco large subunit
ROS	reactive oxygen species
Rubisco	ribulose-1,5-bisphosphate carboxylase
SDS-PAGE	sodium dodecyl sulfate–polyacrylamide gel electrophoresis
SOD	superoxide dismutase
TBA	thiobarbituric acid
TCA	trichloroacetic acid
TEM	transmission electron microscopy
THF	tetrahydrofuran
